# B Cell Tetherin: A Flow Cytometric Cell‐Specific Assay for Response to Type I Interferon Predicts Clinical Features and Flares in Systemic Lupus Erythematosus

**DOI:** 10.1002/art.41187

**Published:** 2020-04-03

**Authors:** Yasser M. El‐Sherbiny, Md Yuzaiful Md Yusof, Antonios Psarras, Elizabeth M. A. Hensor, Kumba Z. Kabba, Katherine Dutton, Alaa A. A. Mohamed, Dirk Elewaut, Dennis McGonagle, Reuben Tooze, Gina Doody, Miriam Wittmann, Paul Emery, Edward M. Vital

**Affiliations:** ^1^ University of Leeds, Leeds, UK, Nottingham Trent University School of Science and Technology, Nottingham, UK, and Mansoura University Mansoura Egypt; ^2^ University of Leeds, Leeds, UK, and NIHR Leeds Biomedical Research Centre, Leeds Teaching Hospitals NHS Trust Leeds UK; ^3^ University of Leeds Leeds UK; ^4^ University of Leeds, Leeds, UK, and Assiut University Assiut Egypt; ^5^ Ghent University Ghent Belgium

## Abstract

**Objective:**

Type I interferon (IFN) responses are broadly associated with autoimmune diseases, including systemic lupus erythematosus (SLE). Given the cardinal role of autoantibodies in SLE, this study was undertaken to investigate whether the findings of a B cell–specific IFN assay correlate with SLE activity.

**Methods:**

B cells and peripheral blood mononuclear cells (PBMCs) were stimulated with type I IFN and type II IFN. Gene expression was analyzed, and the expression of pathway‐related membrane proteins was determined. A flow cytometry assay for tetherin (CD317), an IFN‐induced protein ubiquitously expressed on leukocytes, was validated in vitro and then clinically against SLE diagnosis, plasmablast expansion, and the British Isles Lupus Assessment Group (BILAG) 2004 score in a discovery cohort (n = 156 SLE patients, 30 rheumatoid arthritis [RA] patients, and 25 healthy controls). A second, longitudinal validation cohort of 80 SLE patients was also evaluated for flare prediction.

**Results:**

In vitro, a close cell‐specific and dose‐response relationship between type I IFN–responsive genes and cell surface tetherin was observed in all immune cell subsets. Tetherin expression on multiple cell subsets was selectively responsive to stimulation with type I IFN compared to types II and III IFNs. In patient samples from the discovery cohort, memory B cell tetherin showed the strongest associations with diagnosis (SLE:healthy control effect size 0.11 [*P* = 0.003]; SLE:RA effect size 0.17 [*P* < 0.001]), plasmablast numbers in rituximab‐treated patients (R = 0.38, *P* = 0.047), and BILAG 2004. These associations were equivalent to or stronger than those for IFN score or monocyte tetherin. Memory B cell tetherin was found to be predictive of future clinical flares in the validation cohort (hazard ratio 2.29 [95% confidence interval 1.01–4.64]; *P =* 0.022).

**Conclusion:**

Our findings indicate that memory B cell surface tetherin, a B cell–specific IFN assay, is associated with SLE diagnosis and disease activity, and predicts flares better than tetherin on other cell subsets or whole blood assays, as determined in an independent validation cohort.

## INTRODUCTION

Type I interferons (IFNs) are a highly pleiotropic group of cytokines that link the innate and adaptive immune systems and play a pivotal role in autoimmune disease 1, 2, 3. All nucleated cells express type I IFN receptors and express a set of IFN‐stimulated genes (ISGs) after exposure to type I IFN 4, 5. Hundreds of effects of type I IFN on various cellular processes, interactions, and disease processes have been described. A challenge in the assessment of type I IFN response in an individual disease is therefore ensuring that the appropriate cellular response can be detected within this complex system.

Type I IFN proteins are unstable in blood and not easily detected even in monogenic interferonopathies with known high type I IFN production, possibly due to their efficient binding to the abundant IFN receptor 6. Type I IFN activity is therefore usually measured using the expression of ISGs in whole blood. We previously analyzed ISG expression in sorted cells from patients with systemic lupus erythematosus (SLE), a prototypic IFN‐mediated disease, and healthy individuals and showed that in both groups, ISG expression was markedly higher in monocytes than in other circulating immune cells. ISG expression in monocytes therefore dominates ISG assays that use unsorted blood 7.

These differing levels of ISG expression in different cell populations may be due to the rate of turnover in each population, their trafficking to sites of higher type I IFN production in inflamed tissues, or priming for type I IFN response by other inflammatory mediators. In autoimmunity, type I IFN assays may have value to predict flares and response to a range of different targeted therapies 8. However, existing whole blood IFN biomarkers show poor or uncertain correlation with disease activity 9, 10, 11.

The measurement of type I IFN using ISG expression in whole blood has 2 key weaknesses with regard to interpreting pathogenic processes. First, changes in expression may reflect the expansion or contraction of certain circulating leukocyte populations 12, 13 that differ in their level of ISG expression. This characteristically occurs in inflammatory diseases. In the case of SLE, lymphopenia is almost universally seen 14. So, any difference in whole blood gene expression may not necessarily indicate a change in the production of or exposure to type I IFN. Second, analyzing whole blood ISG expression does not allow the detection of key pathogenic processes among the noise of other, less relevant, effects of type I IFN on biology. For example, B cells are a key mediator in SLE 15, 16. Type I IFN stimulates B cells to differentiate into plasmablasts, which are expanded in SLE and correlate with disease activity 17, 18. We previously demonstrated that the rate of plasmablast regeneration after rituximab treatment predicts clinical outcome 19. We also previously showed that type I IFN imprints plasma cells for the secretion of the proinflammatory molecule ISG‐15 17. Assessment of type I IFN activity in unsorted blood gives limited information about the degree to which B cells have specifically been stimulated by type I IFN. Further, gene expression assays do not prove that a phenotypic change in target cells has occurred—there has been no widely used biomarker for IFN response at the protein level. This may be one reason why some patients classified as having a low IFN signature have responded well to IFN‐blocking therapy 20.

In order to resolve these problems, we developed a flow cytometry assay that allows measurement of type I IFN response in individual cells without the need for cell sorting. We measured the expression of tetherin (also known as bone marrow stromal antigen 2 [BST‐2]; CD317), a glycosyl phosphatidylinositol–anchored protein with a unique topology that is ubiquitously expressed on the surface of nucleated cells. This molecule is prominent in viral immunology and encoded by a commonly measured ISG expressed in all leukocytes 4, 5, 21, 22, 23. Unlike most ISGs, *BST2* encodes a cell surface protein and can be easily measured in patient samples by flow cytometry. Sialic acid–binding Ig‐like lectin 1 (Siglec‐1) is another flow cytometry type I IFN biomarker that has been described previously 24, 25. However, Siglec‐1 is only expressed on monocytes so resolves the issue of changes in the size of cell populations but does not allow interrogation of type I IFN responses in individual cells subsets, including the key B cell populations that are strongly linked to clinical and experimental disease 26, 27, 28.

We hypothesized that a dominant pathogenic role of type I IFN in SLE is its effect on B cells, promoting plasmablast differentiation and clinical disease. Our reasons for addressing B cells as a particular cell of interest in SLE were: 1) SLE is associated with autoantibodies, which are made by B cells; 2) there are a number of susceptibility loci for SLE in genes with important roles in B cell signaling and function, such as *LYN*,* BLK*,* BANK1*,* PTPN22*,* TNFAIP3*, and *TNIP1* 29; and 3) the only targeted therapy licensed for SLE targets B cells specifically. Using in vitro stimulation and sorted cells from SLE patients and healthy individuals, we showed that tetherin accurately captures cell‐specific responses to type I IFN. A crucial issue in biomarker research is demonstrating that biomarkers are predictive, correlate with a range of outcomes, and can be reproduced in validation studies. In our study, longitudinal analysis of discovery and validation cohorts showed that memory B cell tetherin levels more accurately correlated with plasmablast expansion and clinical features of disease, and predicted flares better, compared to monocyte tetherin or whole blood ISG expression.

## PATIENTS AND METHODS

The discovery cohort included 156 consecutive SLE patients, 25 age‐matched healthy controls, and 30 patients with active rheumatoid arthritis (RA) as non‐SLE inflammatory disease controls. The RA patients were positive for anti–citrullinated protein antibody, negative for antinuclear antibody (ANA), and had a mean Disease Activity Score in 28 joints of 3.9 (95% confidence interval [95% CI] 3.23–4.56). An independent validation cohort consisted of 80 SLE patients recruited and studied longitudinally (n = 236 SLE patients total). SLE disease activity was assessed at the time of sampling using the British Isles Lupus Assessment Group 2004 (BILAG 2004) score 30. Patients in the validation cohort were also followed up for subsequent flare (a BILAG score of A or B). SLE patient demographics and disease activity are shown in Supplementary Table 1, available on the *Arthritis & Rheumatology* web site at http://onlin​elibr​ary.wiley.com/doi/10.1002/art.41187/​abstract. Patients with acute or chronic viral infection at the time of blood sampling were excluded from this study.

All individuals provided informed written consent, and the study was carried out in compliance with the Declaration of Helsinki. The patient blood samples used for this study were obtained with ethics approval (REC 10/H1306/88, National Research Ethics Committee Yorkshire and Humber–Leeds East), and healthy control participant peripheral blood samples were obtained under study number 04/Q1206/107. All experiments were performed in accordance with relevant guidelines and regulations. The University of Leeds was contracted with administrative sponsorship. Additional details are included in the Supplementary Methods, available on the *Arthritis & Rheumatology* web site at http://onlin​elibr​ary.wiley.com/doi/10.1002/art.41187/​abstract, and in a previously published methodology article 7.

## RESULTS

### BST‐2/tetherin as a cell‐specific phenotypic biomarker of type I IFN response

Global gene expression profiles have shown that many ISGs are responsive to both IFNα and IFNγ, while other ISGs respond specifically to IFNα 7, 17. We therefore tested the effect of IFNα (type I IFN) and IFNγ (type II IFN) on 31 of the most commonly reported ISGs. TaqMan quantitative polymerase chain reaction (qPCR) analysis of B cells in vitro was performed as previously described 17 (Supplementary Figure 1, available on the *Arthritis & Rheumatology* web site at http://onlin​e​libr​ary.wiley.com/doi/10.1002/art.41187/​abstract). In vitro stimulation confirmed that *BST2* was in the group of ISGs predominantly responsive to type I IFN.

**Figure 1:**
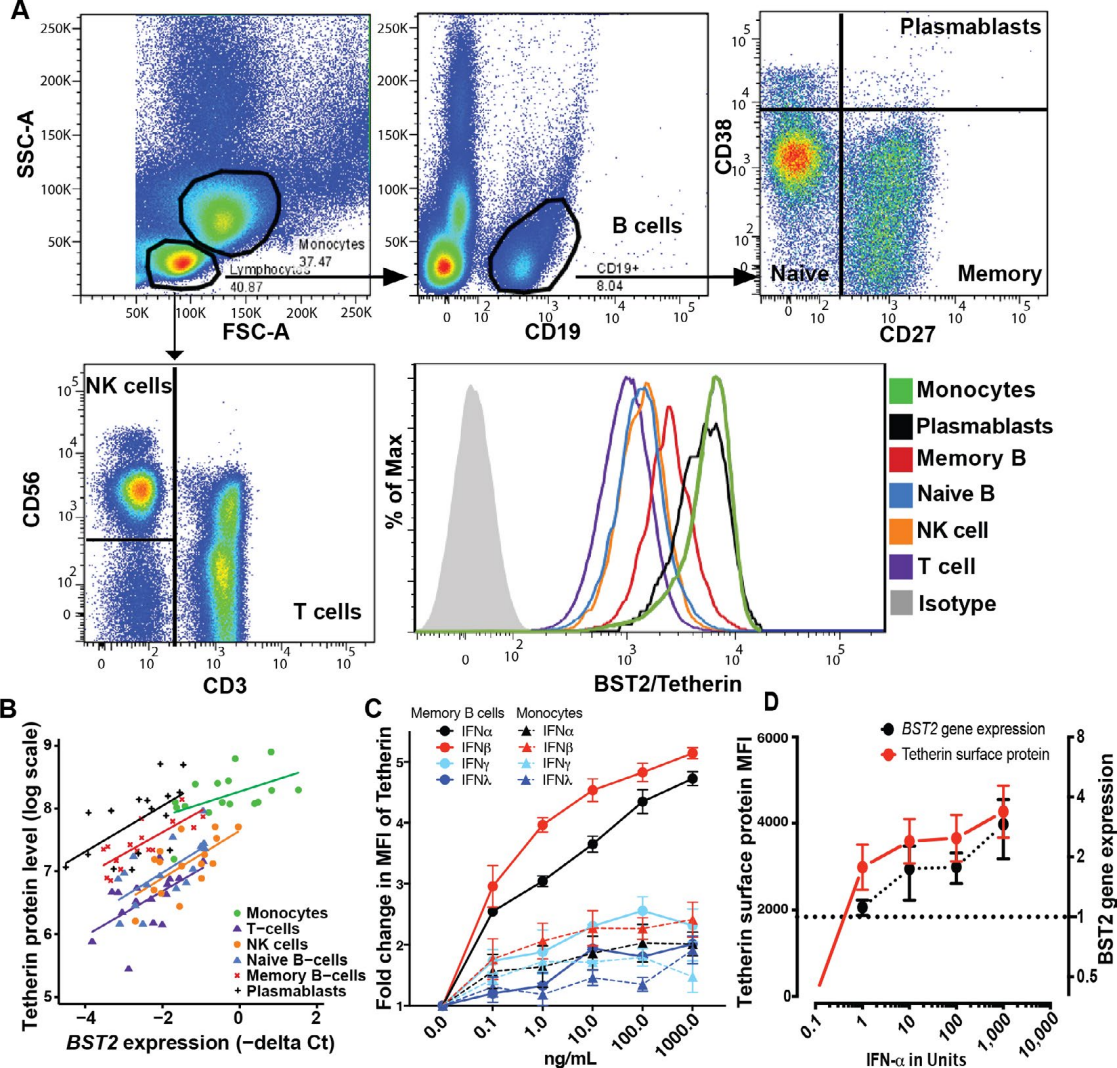
Tetherin is a scalable cell‐specific measure of type I interferon (IFN) response. **A**, Gating strategy for flow cytometric assessment of tetherin on immune cell subsets. A representative flow cytometry plot of tetherin protein expression on individual immune cell subsets is shown. FSC‐A and SSC‐A were used to define lymphocytes and monocytes. B cells were defined as CD19+ lymphocytes and subdivided into naive, memory, and plasmablast subsets using CD27 and CD38. T cells were defined as CD3+, and natural killer (NK) cells were defined as CD3–CD56+ lymphocytes. The mean fluorescence intensity (MFI) of bone marrow stromal antigen 2/tetherin for each cell subset compared to isotype control is shown. **B**, Correlation of tetherin protein level with *BST2* gene expression for the indicated immune cell subsets. In order to validate tetherin as a cell‐specific marker, tetherin protein expression was compared with expression of its gene *BST2* in various immune cell subsets in systemic lupus erythematosus patients and healthy controls. Cell surface tetherin protein levels were determined in unsorted peripheral blood mononuclear cells (PBMCs) by flow cytometry, and *BST2* gene expression data were determined by quantitative polymerase chain reaction of cells sorted by fluorescence‐activated cell sorting. There was a strong correlation between gene expression and protein level within each subset, allowing differences in IFN‐stimulated gene expression between cell subsets to be measured without cell sorting (for monocytes, R = 0.47, *P* = 0.064; for T cells, R = 0.61, *P* = 0.012; for NK cells, R = 0.63, *P* = 0.008; for naive B cells, R = 0.63, *P* = 0.009; for memory B cells, R = 0.78, *P* = 0.001; and for plasmablasts, R = 0.58, *P* = 0.018). **C**, Dose‐dependent response of memory B cell tetherin and monocyte tetherin to IFN. Healthy control PBMCs (n = 3 samples) were stimulated with increasing doses of IFNα, IFNβ, IFNγ, and IFNλ, and tetherin MFI was determined by flow cytometry. **D**, Tetherin protein levels and *BST2* gene expression levels in sorted B cells stimulated in vitro with increasing doses of IFNα and evaluated by flow cytometry. There was a parallel increase in each marker. Dotted line indicates a 1‐fold increase in *BST2* gene expression. In **C** and **D**, values are the mean ± SD.

For this reason, we used multiparameter flow cytometry to detect and quantify tetherin on peripheral blood mononuclear cells (PBMCs), as described in the Supplementary Methods (available on the *Arthritis & Rheumatology* web site at http://onlin​elibr​ary.wiley.com/doi/10.1002/art.41187/​abstract). We used a gating strategy that allowed us to define T cells, natural killer (NK) cells, and monocytes as well as the B cell subsets naive B cells, memory B cells, and plasmablasts. For each of these populations, the mean fluorescence intensity (MFI) of tetherin compared to isotype control was determined (Figure 1A). We compared cell surface tetherin protein levels, determined by flow cytometry, with *BST2* gene expression, determined by qPCR, for these 6 cell subsets sorted by fluorescence‐activated cell sorting from 10 SLE patients and 6 healthy controls. *BST2* gene expression levels were substantially positively correlated with cell surface tetherin protein levels within each of the subsets (Figure 1B). These data confirm that varying levels of tetherin/*BST2* expression between cell subsets and differences between individuals may be captured using flow cytometry without the need for cell sorting. Furthermore, we compared tetherin and Siglec‐1 MFI on monocytes, B cells, and T cells in samples from 25 SLE patients and 5 healthy controls. We confirmed that tetherin correlated with Siglec‐1 on monocytes only because other cell subsets lacked Siglec‐1 expression (Supplementary Figure 2, available on the *Arthritis & Rheumatology* web site at http://onlin​elibr​ary.wiley.com/doi/10.1002/art.41187/​abstract).

### Dose response of tetherin to type I IFN, type II IFN, and type III IFN

We tested the dose‐response relationship of tetherin to IFNα, IFNβ (both type I IFN), IFNγ (type II IFN), and IFNλ (type III IFN) on all circulating cell subsets. Healthy control PBMCs were stimulated for 48 hours with doses of 0.1–1,000 ng/ml and then analyzed by flow cytometry. Interestingly, memory B cell tetherin MFI was most responsive to increasing doses of IFNα and IFNβ, and showed more modest responses to IFNγ and IFNλ. Although monocytes had the highest expression of tetherin in patient samples and the highest basal expression in unstimulated healthy control PBMCs, they showed much lower fold change in tetherin response to type I IFN stimulation (Figure 1C). Furthermore, purified B cell response curves for *BST2* gene expression and tetherin protein MFI revealed a closely matched dose‐response to IFNα (Figure 1D). We concluded that tetherin MFI determined by flow cytometry could accurately measure change in the expression of *BST2* in response to type I IFN and could be used to capture type I IFN exposure in a dose‐ and cell‐specific manner.

### B cell surface tetherin protein levels best demonstrate disease‐associated IFN response in SLE

We next compared tetherin protein expression in immune cell subsets in SLE patients and healthy controls to determine which cell subset best demonstrates disease‐associated change in IFN response. Using all discovery cohort data, tetherin protein levels were compared between the different cell subsets in SLE patients and healthy controls by flow cytometry (Table 1).

**Table 1:** Tetherin levels in cell subsets in SLE patients and healthy controls^a^

	Mean MFI tetherin protein levels in SLE patients (n = 113)^b^	Within SLE, between cell subset		Mean MFI tetherin protein levels in healthy controls (n = 17)^b^	Between group (SLE:healthy controls)		Between group, between cell subset	
		Ratio (90% CI)	*P*		Ratio (90% CI)	*P*	Ratio (90% CI)	*P*
All subjects								
Monocytes	3,388	Reference		2,837	1.19 (0.91–1.58)	0.293	Reference	
T cells	687	0.20 (0.19–0.22)	<0.001	475	1.45 (1.17–1.79)	0.005	1.21 (1.00–1.46)	0.092
NK cells	1,129	0.33 (0.31–0.36)	<0.001	824	1.37 (1.09–1.72)	0.024	1.15 (0.94–1.40)	0.258
Naive B cells	1,118	0.33 (0.30–0.36)	<0.001	712	1.57 (1.22–2.03)	0.004	1.32 (1.03–1.68)	0.062
Memory B cells	1,586	0.47 (0.43–0.50)	<0.001	1,033	1.53 (1.23–1.92)	0.002	1.29 (1.04–1.58)	0.046
Plasmablasts	2,650	0.78 (0.72–0.84)	<0.001	1,813	1.46 (1.15–1.86)	0.009	1.22 (0.99–1.51)	0.115
Rituximab‐naive only^c^								
Monocytes	3,206	Reference		2,949	1.09 (0.80–1.48)	0.657	Reference	
T cells	666	0.21 (0.19–0.23)	<0.001	494	1.35 (1.07–1.70)	0.034	1.24 (1.01–1.52)	0.080
NK cells	1,068	0.33 (0.31–0.36)	<0.001	857	1.25 (0.98–1.58)	0.129	1.15 (0.93–1.41)	0.271
Naive B cells	1,132	0.35 (0.32–0.39)	<0.001	740	1.53 (1.20–1.95)	0.004	1.41 (1.15–1.73)	0.006
Memory B cells	1,574	0.49 (0.45–0.53)	<0.001	1,074	1.47 (1.17–1.83)	0.005	1.35 (1.11–1.64)	0.013
Plasmablasts	2,597	0.81 (0.74–0.89)	<0.001	1,885	1.38 (1.08–1.76)	0.033	1.27 (1.02–1.57)	0.068

a Tetherin cell protein data were natural log–transformed prior to analysis. The back‐transformed results represent the ratio of the value for each cell subset relative to the value for monocytes within the group of patients with systemic lupus erythematosus (SLE), and the ratio of the value for each cell subset in SLE patients relative to that in healthy controls. Interaction ratios (between group, between cell subset) are the ratio of the extent of the difference in the value for each cell subset relative to monocytes between SLE patients and healthy controls. MFI = mean fluorescence intensity; 90% CI = 90% confidence interval; NK = natural killer.

b Adjusted for age.

c n = 76 patients with SLE.

Tetherin levels differed significantly between cell subsets within the group of SLE patients and were highest in monocytes. Tetherin levels in T cells were 20% of the levels in monocytes, and tetherin levels in plasmablasts were 78% of the levels in monocytes (both *P* < 0.001). A comparison of the SLE and healthy control groups showed that monocyte tetherin MFI in SLE patients did not differ significantly from that in healthy controls (SLE:healthy control ratio 1.19; *P* = 0.293), whereas a significantly higher level of tetherin was seen in all other subsets in SLE patients versus healthy controls (ratios 1.37–1.57; all *P* < 0.05). When the between‐group ratio for each of the other cell subsets was compared to that for monocytes, the greatest difference was seen for memory B cell tetherin levels, which were increased in SLE patients (*P* = 0.046).

Rituximab treatment could confound accurate measurement of the B cell phenotype in the SLE patients. We therefore repeated these analyses in rituximab‐naive patients (Table 1). In rituximab‐naive patients (n = 76), the largest disease‐associated increases in tetherin expression versus healthy controls were seen for naive B cells (ratio 1.53) and memory B cells (ratio 1.47). These ratios for naive and memory B cells were significantly different from that for monocytes (*P* = 0.006 and *P* = 0.013, respectively). These results indicate that differences in IFN response between cell subsets at the protein level are clinically relevant, and that B cell tetherin is the most clinically relevant parameter.

### Tetherin and IFN gene expression assays

Overall comparisons of tetherin levels measured on memory B cells and 2 validated IFN gene expression scores are shown in Supplementary Figures 3 and 4, available on the *Arthritis & Rheumatology* web site at http://onlin​elibr​ary.wiley.com/doi/10.1002/art.41187/​abstract. As expected, given the difference in cell populations analyzed, there was a significant correlation but a degree of disagreement between these assays.

### Clinical validation of the tetherin IFN assay: diagnosis.

**Figure 2:**
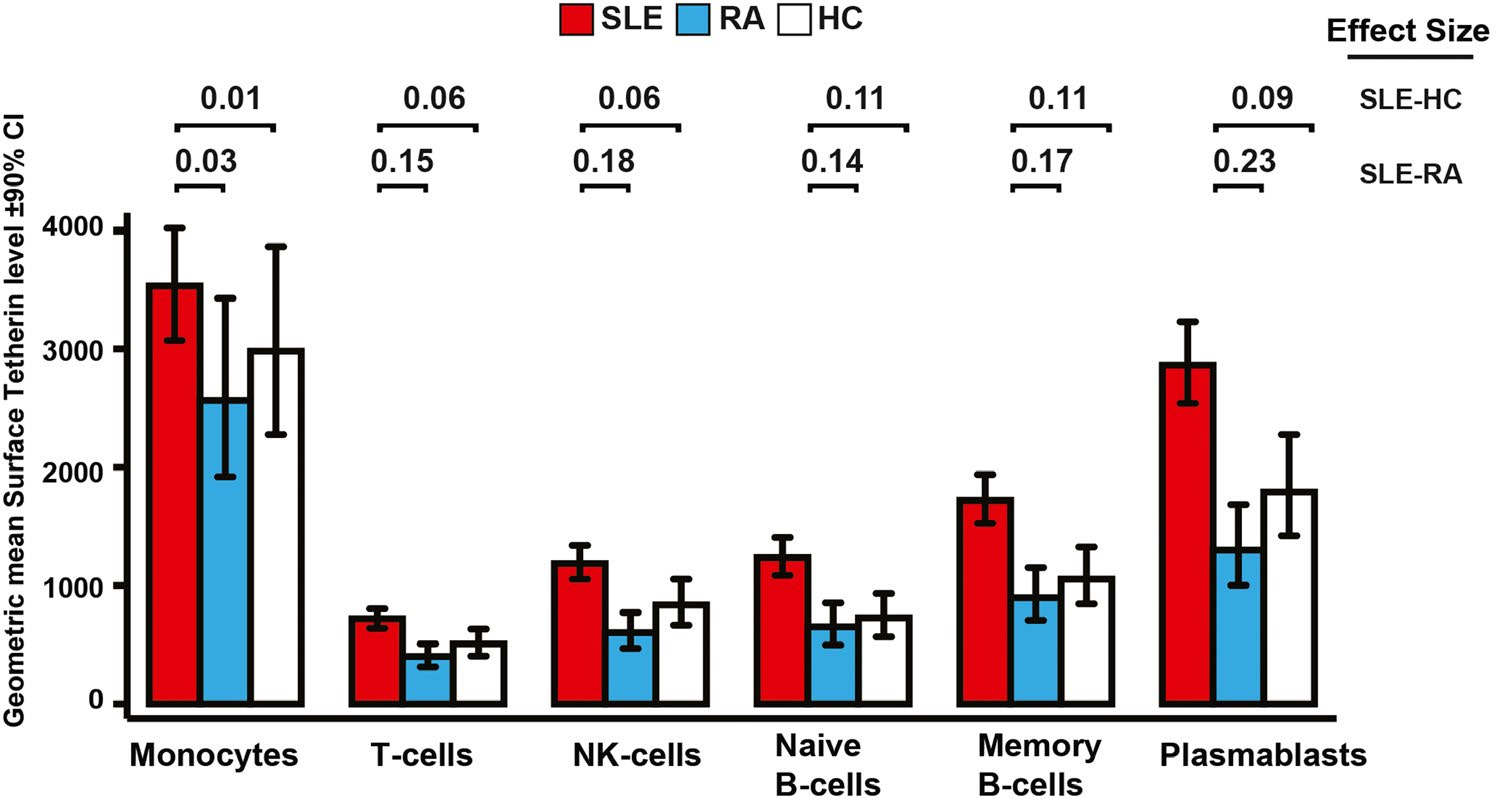
Performance of the tetherin interferon (IFN) flow cytometry assay in discriminating patients based on diagnosis. Age‐adjusted differences in tetherin levels on the indicated cell subsets between patients with systemic lupus erythematosus (SLE), patients with active rheumatoid arthritis (RA) (with a Disease Activity Score in 28 joints of >3.2), and healthy controls (HCs) are shown. Cell surface bone marrow stromal antigen 2/tetherin protein levels were determined by flow cytometry of peripheral blood mononuclear cells. Effect sizes (partial η^2^) indicate the degree to which variables differed between groups. We considered an effect size of ≤0.01 to be small, ~0.06 to be medium, and ≥0.14 to be large 31. Bars show the mean and 90% confidence interval (90% CI). NK = natural killer. Color figure can be viewed in the online issue, which is available at http://onlinelibrary.wiley.com/doi/10.1002/art.41187/abstract.

We evaluated the performance of the tetherin flow cytometry assay in distinguishing between patients diagnosed as having SLE, those diagnosed as having active RA, and healthy controls. Given our previous results, for this analysis we included only rituximab‐naive patients controlled for age (Figure 2). The full statistical table is shown in Supplementary Table 2, available on the *Arthritis & Rheumatology* web site at http://onlin​elibr​ary.wiley.com/doi/10.1002/art.41187/​abstract. An effect size of ≤0.01 was considered small, ~0.06 medium, and ≥0.14 large, as described by Cohen 31. Tetherin data revealed a marked difference between cell subsets. Monocyte tetherin levels did not differentiate SLE patients from healthy controls at all, with a ratio of 1.19 (90% CI 0.87–1.61) and an effect size of 0.01. T cells and NK cells had moderate effect sizes of 0.06 each. However, naive and memory B cell subsets had medium to large effect sizes of 0.11, with ratios of 1.63 (90% CI 1.26–2.11) and 1.59 (90% CI 1.21–2.09), respectively. For memory B cells, the SLE:healthy control effect size was 0.11 (*P* = 0.003), and the SLE:RA effect size was 0.17 (*P* < 0.001).

Tetherin levels differentiated SLE from other inflammatory disease when compared to active RA. Monocyte tetherin levels had no diagnostic function, with a ratio of 1.37 (90% CI 1.00–1.88) and an effect size of 0.03. However, all other cell subsets had large effect sizes, ranging from 0.14 to 0.23, the effect size for plasmablasts (ratio 2.20 [90% CI 1.66–2.93]).

### Clinical validation of IFN assays: disease activity and autoantibodies

For disease activity, we investigated the association between the number of active organ systems (BILAG domains scored A, B, or C) per patient compared to tetherin levels on cell subsets as well as our recently described IFN score A, which comprises 12 type I IFN–selective ISGs 7. We controlled for age in all SLE patients (164 observations in 124 patients). The number of active domains was categorized as 0 (n = 22), 1 (n = 54), 2 (n = 57), or ≥3 (n = 31).

At the 10% level of significance, disease activity was associated with IFN score A (R^2^ = 0.08, *P =* 0.027) and tetherin surface expression on T cells (R^2^ = 0.07, *P =* 0.007), NK cells (R^2^ = 0.09, *P =* 0.001), memory B cells (R^2^ = 0.09, *P =* 0.006), and plasmablasts (R^2^ = 0.06, *P =* 0.020). The degree of association was weaker, and hence not significant, for monocytes (R^2^ = 0.04, *P =* 0.179) and naive B cells (R^2^ = 0.04, *P =* 0.103).

**Figure 3:**
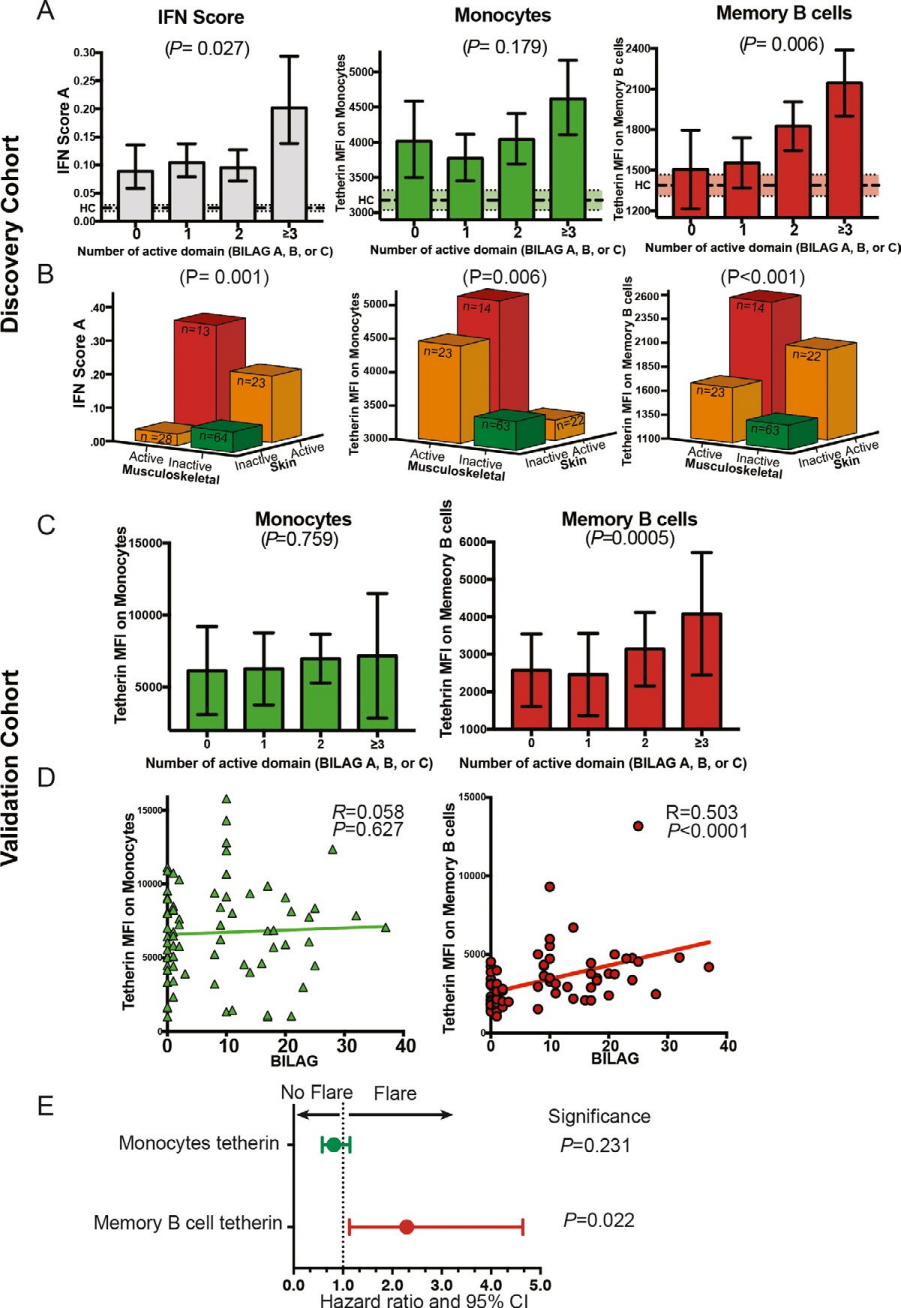
Association between interferon (IFN) assays and disease activity in systemic lupus erythematosus (SLE). **A**, Association between different IFN assays (IFN score A, monocyte tetherin levels, and memory B cell tetherin levels) and the number of organ systems (domains) with active disease in the discovery cohort (164 observations in 124 SLE patients). IFN score A was increased in patients with ≥3 active domains, but not in patients with 1 or 2 active domains, compared to those with 0 active domains (remission). Tetherin mean fluorescence intensity (MFI) measured on memory B cells demonstrated a more consistent stepwise increase with increasing disease activity. Bars show the mean and 90% confidence interval (90% CI), calculated using the 2^−ΔCt^ method (i.e., taller bars represent higher expression). Broken lines and shaded areas represent the mean and 90% CI in healthy controls (HCs; n = 23). **B**, Association between different IFN assays and musculoskeletal and mucocutaneous disease activity. Disease activity was defined as active (British Isles Lupus Assessment Group [BILAG] score of A or B) or inactive (BILAG score of D or E). Patients with activity in other organs were excluded. For IFN score A, there were inconsistent relationships with disease activity, with an increase with skin involvement, but not musculoskeletal involvement alone. For monocyte tetherin levels, increased protein expression was seen with musculoskeletal disease activity but not with skin activity alone. Tetherin levels measured on memory B cells demonstrated a consistent relationship with both common types of clinical disease. Bars show the median. **C**, Association between different IFN assays (monocyte tetherin levels and memory B cell tetherin levels) and the number of organ systems with active disease in the validation cohort. Results were similar to those for the discovery cohort, shown in **A**. Bars show the mean ± SD (n = 80 patients). **D**, Scatterplots showing association between overall disease activity (BILAG global score) and tetherin levels. There was a significant association between BILAG global score and memory B cell tetherin levels but not monocyte tetherin levels. **E**, Relationship between tetherin levels and SLE disease flare. Memory B cell tetherin levels were significantly predictive of subsequent clinical flare (hazard ratio [HR] 2.290 [95% CI 1.013–4.644]; *P =* 0.022), while monocyte tetherin levels were not (HR 0.814 [95% CI 0.580–1.141]; *P* = 0.231).

For IFN score A, the relationship with disease activity was not linear. The only significant association between the score and disease activity was attributable to patients with the most severely active disease (≥3 domains). A similar, although not significant, pattern was observed for monocyte tetherin levels. In contrast, there was a linear relationship between memory B cell tetherin levels and disease activity, with a stepwise increase in tetherin expression for each increase in the number of active domains (Figure 3A). We did not expect a strong correlation between memory B cell tetherin levels and IFN score in unsorted PBMCs. Since memory B cells are only ~2% of PBMCs, these assays do not measure the same biologic effect. We found a moderate correlation (Spearman's R = 0.356, *P* < 0.0001) (Supplementary Figure 3, available on the *Arthritis & Rheumatology* web site at http://onlin​elibr​ary.wiley.com/doi/10.1002/art.41187/​abstract).

To investigate whether the difference between IFN assays was due to the type of organ system affected, we analyzed the 2 most commonly affected domains (mucocutaneous and musculoskeletal) in combination, excluding patients with activity (BILAG scores of A, B, or C) in any of the other domains. Although there was a significant relationship between each IFN assay and overall disease activity, the relationship with IFN assays varied between these 2 organ systems (Figure 3B). For IFN score A, increased expression was only seen with mucocutaneous disease activity. For monocyte tetherin levels, increases were observed only in patients with musculoskeletal disease activity. This finding may explain why this assay does not show a linear relationship with disease activity. However, memory B cell tetherin had a more consistent relationship with disease activity in both organ systems. Tetherin levels were lowest in patients in clinical remission, higher in patients with active disease in a single organ, and highest in patients with active disease in both organs.

The numbers of patients with other active organ domains were more limited. Of patients with no activity in other domains, 12 had active hematologic disease (BILAG score of A or B) (immune‐mediated hemolysis or thrombocytopenia). Memory B cell tetherin MFI in the patients with active hematologic disease versus those with inactive disease was 1,954 versus 1,494, respectively (*P =* 0.005). Eight patients had active renal disease. Comparing these 8 patients with active renal disease to patients with inactive disease also revealed a significant increase in memory B cell tetherin levels (MFI 2,625 versus 1,562; *P =* 0.005). Tetherin levels were not associated with glucocorticoid use (Supplementary Figure 5, available on the *Arthritis & Rheumatology* web site at http://onlin​elibr​ary.wiley.com/doi/10.1002/art.41187/​abstract). In our rituximab‐naive patients, there was a positive correlation between memory B cell tetherin expression and autoantibodies summarized as the number of extractable nuclear antigen subtypes (R = 0.412, *P* = 0.0001) (Supplementary Figure 6, available on the *Arthritis & Rheumatology* web site at http://onlin​elibr​ary.wiley.com/doi/10.1002/art.41187/​abstract).

For additional comparison to alternative IFN assays, we classified patients as type I IFN positive or type I IFN negative according to a 5‐gene IFN signature, as described by Higgs et al 32. Results are shown in Supplementary Figure 7, available on the *Arthritis & Rheumatology* web site at http://onlin​elibr​ary.wiley.com/doi/10.1002/art.41187/​abstract. The majority of the SLE patients were in the type I IFN–positive subgroup. As expected, this subgroup had worse BILAG disease activity (*P* = 0.016). To determine whether tetherin expression gave additional information in comparison to the gene expression status, we retested the association of tetherin levels with BILAG scores within the type I IFN–positive subgroup. We still found a significant association between tetherin expression and disease activity (Spearman's R = 0.321, *P* = 0.038), which could not be measured using the more standard assay. This finding indicates that memory B cell tetherin gives additional clinically relevant information compared to the IFN signature alone.

### Clinical validation of IFN assays: plasmablasts

Last, in the discovery cohort, we used the plasmablast count to represent current B cell activity and differentiation. Type I IFN is known to promote the differentiation of memory B cells into plasmablasts 33. We have previously shown that an early rapid population of plasmablasts after rituximab treatment led to an early clinical relapse 19, 34. We hypothesized that the memory B cell tetherin level would correlate with circulating plasmablast numbers after rituximab treatment, reflecting an increased rate of differentiation secondary to type I IFN. The results are shown in Table 2. In rituximab‐naive patients, no relationship was found between any tetherin IFN assay and plasmablast count. In patients who had received rituximab treatment there was no correlation between monocyte, NK, or T cell tetherin levels and plasmablast numbers, but memory B cell tetherin levels were significantly correlated with plasmablast numbers (Spearman's R = 0.38, *P* = 0.047) as well as inversely correlated with time to clinical relapse (R = 0.623, *P* = 0.022). To further explore whether tetherin surface protein expression was associated with the induction of relevant pathogenic pathways in B cells, we evaluated 2 transcripts for downstream plasmablast function: *IgJ* for antibody synthesis in all samples, and *ISG15* for ISG‐15 protein secretion in sorted memory B cells. Both of these transcripts showed a significant correlation with flow cytometric measurement of memory B cell tetherin MFI (Supplementary Figure 8, available on the *Arthritis & Rheumatology* web site at http://onlinelibrary.wiley.com/doi/10.1002/art.41187/abstract).

**Table 2:** Association between candidate IFN assays and plasmablast level following B cell depletion therapy in SLE patients^a^

	Plasmablast count before rituximab treatment (n = 50)	Plasmablast count after rituximab treatment (n = 28)
IFN score A	–0.11	0.24
Tetherin protein level		
Monocytes	–0.08	0.20
T cells	–0.16	0.32
NK cells	–0.14	0.05
Naive B cells	–0.04	0.30
Memory B cells	0.07	0.38^b^

a Values are Spearman's rank correlation coefficient. Interferon (IFN) score A was measured on unsorted peripheral blood mononuclear cells, and tetherin mean fluorescence intensity (MFI) on each cell subset was analyzed by flow cytometry. SLE = systemic lupus erythematosus; NK = natural killer.

b *P* = 0.047 for correlation between plasmablast count and tetherin MFI.

### Independent validation cohort

The independent validation cohort consisted of an additional 80 patients with SLE who were recruited and studied prospectively. Memory B cell and monocyte tetherin levels were measured using fresh lysed whole blood in an independent accredited diagnostic laboratory. Disease activity was measured at the time of sampling using the BILAG 2004. Patients were followed up for subsequent flare (a BILAG score of A or B).

We found a similar relationship between tetherin levels and disease activity as in our discovery cohort. There was a significant relationship between the number of organ domains with active disease and memory B cell tetherin levels (*P =* 0.0005) but no relationship with monocyte tetherin levels (*P =* 0.759) (Figure 3C). There was a significant association between global BILAG score and memory B cell tetherin levels (Spearman's R = 0.503, *P* < 0.0001) but no association with monocyte tetherin levels (R = 0.058, *P =* 0.627) (Figure 3D). Additionally, in this cohort we demonstrated that in patients in clinical remission at the time of sampling (n = 36), memory B cell tetherin levels predicted time to clinical flare. In a multivariable Cox regression analysis including memory B cell tetherin level, monocyte tetherin level, and age, memory B cell tetherin level was a significant predictor of subsequent BILAG A/B flare (hazard ratio [HR] 2.290 [95% CI 1.013–4.644]; *P =* 0.022). Monocyte tetherin level did not significantly predict flare (HR 0.814 [95% CI 0.580–1.141]; *P* = 0.231) (Figure 3E). In conclusion, we independently confirmed that disease activity is related to type I IFN response in memory B cells measured using tetherin, and further, that this is predictive of clinical outcome.

## DISCUSSION

In this study we demonstrated the value of a novel cell‐specific biomarker based on the IFN‐inducible protein tetherin, using in vitro methods and human clinical studies. We showed that flow cytometric measurement of memory B cell surface tetherin levels captured cell‐specific type I IFN response, was responsive to increasing doses of type I IFN, and had a strong and consistent relationship with disease activity, B cell activity, and time to flare in 2 cohorts of SLE patients. These results are important because type I IFN and B cells play a role in many autoimmune diseases, and their measurement has the potential to stratify outcomes and use of therapies, though previous studies have yielded conflicting results 35.

Better biomarkers are needed in SLE. European League Against Rheumatism treat‐to‐target recommendations advise treating to a target of low disease activity, while minimizing exposure to glucocorticoids 36. Predictors of a severe disease trajectory or flares are needed to achieve this goal. Response to conventional and targeted therapies in SLE and related diseases is variable, and reclassification of autoimmune diseases according to pathogenic mechanisms instead of clinical features has been proposed 35.

The crucial role of type I IFN in the pathogenesis of SLE and related diseases is indicated by genetic susceptibility and monogenic interferonopathies as well as evidence of overexpression 35. As such, it has face validity as a stratification biomarker. Existing studies indicate the potential value of measuring type I IFN for diagnosis and prediction of flares. Type I IFN biomarkers may also predict clinical response to tumor necrosis factor blockade, B cell depletion, and type I IFN blockade in RA and SLE 35.

Nevertheless, there are limitations to previous approaches to measuring type I IFN activity, and some previous results have been contradictory. Direct measurement of type I IFN protein is limited by the number of different ligands and instability in serum, with most cell types expressing the type I IFN receptor. A recent improvement was the use of single‐molecule arrays (Simoa). The higher sensitivity of Simoa allows for reliable measurement of IFNα 6. However, this assay is currently expensive and limited in availability and has not been validated against clinical outcomes. For ISG expression–based methods, another issue is the effect of other IFN subtypes or other inflammatory mediators. ISGs are known to fall into distinct subsets, which may be due to the effect of type II IFN 7, 9. We previously showed that there are different patterns of ISG expression in different autoimmune diseases. In the present study we confirmed that tetherin is selectively responsive to type I IFN, and we included ANA‐negative RA patients as inflammatory disease controls. (We did not see any elevation of tetherin levels in our RA patients as others have reported for an IFN signature, but this difference may be due to our selection of only ANA‐negative cases rather than differences in the biomarkers).

While candidate biomarker discoveries in autoimmunity are numerous, a significant challenge is validation in clinically relevant contexts 37. An important aspect of our work is the degree of preclinical and clinical validation. We used 2 methods of validation to demonstrate that tetherin reflects cellular response to type I IFN. We demonstrated a correlation with existing validated IFN assays. However, such concurrent validity studies are limited by the potential imprecision of the IFN scores. These scores may be affected by changes in the cellular composition of the sample or other subtypes of IFNs. Moreover, tetherin assesses the response to IFN of a specific cell subset (we have shown memory B cells), while IFN scores assess a mixed population of cells and will be influenced by other cell types. For these reasons, the more important method of demonstrating that tetherin reflects cellular response to type I IFN is through in vitro stimulation assays. We showed that tetherin has a dose‐dependent response to type I IFN in multiple cell subsets, far exceeding response to type II IFN. Our data therefore demonstrate good face and construct validity, as well as concurrent and prospective criterion validation and feasibility in a routine clinical setting. We also present validation against a range of different clinical and longitudinal end points.

Cell‐specific measurement based on flow cytometry has been demonstrated previously using expression of Siglec‐1, another cell surface protein convenient for flow cytometry that is expressed by monocytes. Monocyte Siglec‐1 expression has been shown to correlate with disease activity as well as predict autoimmune congenital heart block 25, 38, 39. This was a significant advance in analysis of IFN status. In the present study we advanced this principle further by using a marker expressed on all circulating cells. Tetherin captures the same information as Siglec‐1 on monocytes, but also evaluates other cell subsets. We have shown that results from these different subsets vary, with the strongest clinical correlation for memory B cells. This method has distinct advantages when there is particular interest in a specific cell population, such as with the B cell–directed therapies rituximab and belimumab in SLE. B cell response to type I IFN is crucial in SLE.

While there were many associations between tetherin protein expression and clinical features of SLE, memory B cell tetherin levels seemed to be particularly important. This marker correlated best with clinical features, and was the only marker to be associated with plasmablast number. After B cell depletion with rituximab, there is a highly variable rate of plasmablast repopulation that predicts clinical relapse. Understanding the determinants of these repopulation patterns may reveal upstream factors controlling B cell autoreactivity. One previous study showed a relationship between serum BAFF titers and the numbers of plasmablasts at relapse 40. However, BAFF may not be the only factor. Type I IFN also promotes B cell activation and differentiation into plasmablasts and plasma cells 28, 41. This may include direct influences; for example, in animal models type I IFN influences B cell receptor– and Toll‐like receptor–mediated response to self nuclear antigen. Our work provides data from human disease to support this observation 42, 43. Additionally, type I IFN induces a plasma cell phenotype that secretes ISG‐15 with additional proinflammatory effects 17. In the present study, we found that memory B cell tetherin levels correlated with plasmablast expansion after rituximab treatment. A plasmablast signature was recently shown to be a strong biomarker for SLE, and we and others previously showed that plasmablast expansion after rituximab was strongly predictive of clinical relapse 19, 44, 45. This was further supported by a correlation between memory B cell tetherin levels and transcripts representing disease‐relevant B cell dysfunction.

The tetherin biomarker has some limitations. First, although this flow cytometry assay avoids confounders that may affect ISG expression scores, analyzing a single IFN‐inducible transcript may be more susceptible to the influence of other inflammatory stimuli, which we cannot exclude based on these results. However, our data comparing SLE to the RA disease control are very consistent with those we observed using IFN scores, with a clear difference in IFN score A and tetherin expression between SLE and RA. Tetherin, like all type I IFN biomarkers, may be influenced by acute or chronic viral infections, which were excluded from this study. It may be more difficult to perform flow cytometry in some situations. However, with widespread use of flow cytometry in cell‐targeted therapies in autoimmunity and oncology, as well as in routine monitoring of HIV, addition of tetherin cell surface staining is a highly cost‐effective test. Tetherin may be analyzed in combination with B cell and plasmablast flow cytometry to stratify both B cell– and type I IFN–blocking therapy.

In summary, we describe measurement of the IFN‐inducible protein tetherin on B cells as a cell‐specific biomarker with a number of advantages and widespread applications in clinical and laboratory research in this rapidly expanding area of immunology.

## AUTHOR CONTRIBUTIONS

All authors were involved in drafting the article or revising it critically for important intellectual content, and all authors approved the final version to be published. Dr. Vital had full access to all of the data in the study and takes responsibility for the integrity of the data and the accuracy of the data analysis.

### Study conception and design

El‐Sherbiny, Emery, Vital.

### Acquisition of data

El‐Sherbiny, Md Yusof, Psarras, Hensor, Kabba, Dutton, Mohamed, Elewaut, McGonagle, Tooze, Doody, Wittmann.

### Analysis and interpretation of data

El‐Sherbiny, Md Yusof, Psarras, Hensor, Kabba, Dutton, Mohamed, Elewaut, McGonagle, Tooze, Doody, Wittmann, Emery, Vital.
